# Safety of Artemisinin Derivatives in the First Trimester of Pregnancy: A Controversial Story

**DOI:** 10.3390/molecules25153505

**Published:** 2020-07-31

**Authors:** Sarah D’Alessandro, Elena Menegola, Silvia Parapini, Donatella Taramelli, Nicoletta Basilico

**Affiliations:** 1 Dipartimento di Scienze Biomediche, Chirurgiche e Odontoiatriche, Università degli Studi di Milano, 20133 Milan, Italy; sarah.dalessandro@unimi.it; 2 Dipartimento di Scienze e Politiche Ambientali, Università degli Studi di Milano, 20133 Milan, Italy; elena.menegola@unimi.it; 3 Dipartimento di Scienze Biomediche per la Salute, Università degli Studi di Milano, 20133 Milan, Italy; silvia.parapini@unimi.it; 4 Dipartimento di Scienze Farmacologiche e Biomolecolari, Università degli Studi di Milano, 20133 Milan, Italy; donatella.taramelli@unimi.it

**Keywords:** artemisinins, safety, pregnancy, embryotoxicity, malaria

## Abstract

Artemisinin combination therapy (ACT) is recommended by the World Health Organization (WHO) as first line treatment for uncomplicated malaria both in adults and children. During pregnancy, ACT is considered safe only in the second and third trimester, since animal studies have demonstrated that artemisinin derivatives can cause foetal death and congenital malformation within a narrow time window in early embryogenesis. During this period, artemisinin derivatives induce defective embryonic erythropoiesis and vasculogenesis/angiogenesis in experimental models. However, clinical data on the safety profile of ACT in pregnant women have not shown an increased risk of miscarriage, stillbirth, or congenital malformation, nor low birth weight, associated with exposure to artemisinins in the first trimester. Although further studies are needed, the evidence collected up to now is prompting the WHO towards a change in the guidelines for the treatment of uncomplicated malaria, allowing the use of ACT also in the first trimester of pregnancy.

## 1. Introduction

Malaria continues to be a major threat in the developing world, with 228 million clinical episodes and 405,000 deaths in 2018 [1]. Most of disease-associated morbidity and mortality occurs in children and pregnant women in sub-Saharan Africa and *Plasmodium (P.) falciparum* is the most common cause. Clinical features of *P. falciparum* infection include asymptomatic parasitemia, uncomplicated malaria and complicated or severe malaria, often lethal. Severe malaria is essentially due to the cytoadherence of infected erythrocytes to the vascular endothelium of vital organs, causing microcirculatory obstruction and the development of a generalized inflammatory response [2]. Malaria during pregnancy can produce harmful effects on both the mother and the foetus, depending on transmission intensity. In high transmission areas the risks of adverse effects are most prominent for women in their first pregnancy. In low transmission areas, where women have low acquired immunity, malaria often develops into severe diseases [3].

**Figure 1:**
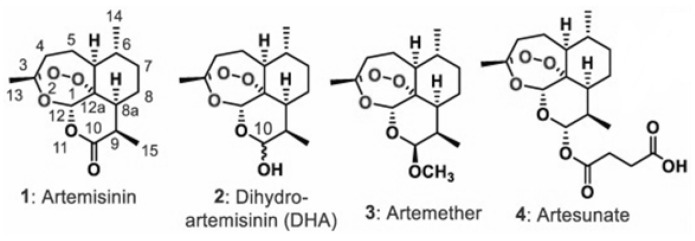
Artemisinin 1 and its derivatives, dihydroartemisinin (DHA) 2, artemether 3, and artesunate 4.

Artemisinin derivatives are the mainstay class of drugs for malaria therapy, showing excellent efficacy in both severe and uncomplicated malaria. Artemisinin (Figure 1) is the active principle extracted from *Artemisia annua* (qinghao), a plant of the *Asteraceae* family, used for ages in traditional Chinese medicine [4]. It is a sesquiterpene lactone with a peroxide bridge essential for the antimalarial effect. To overcome the poor solubility of artemisinin and, thus, the formulation issues, semisynthetic derivatives of artemisinin with improved pharmacological properties, including artemether, artesunate and dihydroartemisinin (DHA), were developed (Figure 1). Artemisinins are safe and well tolerated drugs, with a very rapid cytotoxic action against all parasite stages, including early ring stage [5,6,7,8]. They are also active against early stage gametocytes, responsible for transmission from humans to mosquitos [9,10]. The large deployment and usage of Artemisinin-based combination therapy (ACT) in endemic settings has contributed significantly to the reduction in mortality and morbidity by malaria over the last decade. ACT is suggested during the second and third trimester. However, based on experimental data, the use of ACT during the first trimester of pregnancy is cautioned for evidences of embryotoxicity [11,12], and WHO guidelines still do not recommend ACT for uncomplicated malaria in the first trimester of pregnancy [13,14].

Several studies recently concluded indicate that artemisinins are safe and could be used also during the first trimester [15]. Therefore, a reexamination of WHO policy is probably necessary.

## 2. Pathophysiology and Epidemiology of Malaria in Pregnancy

Children and pregnant woman are particularly susceptible to severe malaria caused by *P. falciparum* infection. In pregnant women, the accumulation of infected erythrocytes in the placenta accounts for low birth weight, infant anaemia and stillbirth [16]. Sequestration of infected erythrocytes in the placenta is due to the expression, on the surface of infected red blood cells, of variant surface antigens (VAR2CSA) able to mediate the adherence to placental chondroitin sulfate A and to other placental receptors [17,18]. This sequestration leads to mononuclear cell recruitment, placental damage, inflammation, clogging and failure to clear parasites through normal immunity [19]. Women during their first pregnancy have increased risk of developing the severe forms of malaria, especially severe anemia, but also acute respiratory distress syndrome, renal failure and cerebral malaria. Malaria during pregnancy is responsible for adverse effects on the fetus, too. Besides stillbirth, whose impact depends on malaria endemicity, low birth weight and preterm delivery are common and are responsible for increased infant mortality [3].

In 2018, about 11 million women were exposed to malaria during pregnancy. Prevalence of exposure to malaria infection during pregnancy was 30% or more in 20 African countries. About 39% of malaria pregnancy cases were in the Democratic Republic of the Congo and Nigeria. WHO analysis showed a positive correlation between maternal anemia and prevalence of exposure to malaria infection during pregnancy [1].

## 3. Current Therapies of Malaria in Pregnancy

### 3.1. Therapy of Malaria in Pregnancy

ACTs are recommended by the World Health Organization (WHO) for adults and children as first line treatment for uncomplicated malaria but in pregnant women ACTs are restricted to the second and third trimester (Table 1). The current treatments with artemether/lumefantrine or dihydroartemisinin/piperaquine have demonstrated themselves to be well tolerated and safe during the second and third trimester of pregnancy in controlled trials. However, as stated above, due to their potential embryotoxicity, ACTs have not been considered safe in the first trimester of pregnancy. As a consequence, the WHO recommends quinine plus clindamycin to treat uncomplicated malaria (Table 1) [13,20]. Intravenous artesunate is, however, the treatment of choice for severe malaria in children and adults, including pregnant women in the first trimester [21]. The primary objective of the treatment of severe malaria in pregnancy is indeed to save the life of the mother and randomized clinical trials showed a significant reduction of mortality with parental artesunate compared to quinine [22,23]. Data on animal models indicate that artemisinins are embryotoxic during a limited time window in the first trimester of pregnancy (corresponding to 4–10 weeks of human gestation), probably acting by altering erythropoiesis and vasculogenesis/angiogenesis [24]. Unfortunately, the information regarding the safety profile of most of the licensed antimalarial treatments in humans during pregnancy is limited since pregnant women are not usually involved in clinical trials during drug development. It is indeed unclear if artemisinin-induced embryotoxicity can occur in human embryos, as well. Current data are incomplete and further studies are needed to determine artemisinin’s safety in the first trimester of pregnancy. However, in the recent years, meta-analysis and observational/prospective studies have been performed, which support the decision of the Malaria Policy Advisory Committee of the WHO to endorse the use of ACTs in the first trimester, although this decision has not been converted to a recommendation in the treatment guidelines, yet [13,25,26,27].

### 3.2. Intermittent Preventive Treatment (IPT)

Intermittent preventive treatment during pregnancy (IPTp) consists of the administration of an antimalarial drug to all pregnant women, independently from malaria diagnosis. The WHO recommends IPTp with sulfadoxine–pyrimethamine (IPTp-SP) in all areas with moderate to high malaria transmission in Africa but not in areas of low transmission level [28]. IPT should be administered at each routine prenatal care visit, starting as early as possible in the second trimester, not during the first trimester. The women should receive at least three doses of SP during pregnancy (IPTp3), with each dose administrated at least 1 month apart. Less than three doses is considered sub-optimal. SP can safely be administered until delivery [29]. Based on currently available evidence, IPTp-SP remains effective in preventing the adverse consequences of malaria on maternal and foetal outcomes even in areas where quintuple mutations linked to SP resistance are prevalent in *P. falciparum.* Therefore, there is a need to continuously monitor the effectiveness of this useful intervention in the light of increasing *P. falciparum* resistance to SP [30]. Pregnant women are regularly given a folic acid supplementation. However, since high doses of folic acid counteract the effect of sulfadoxine–pyrimetamine, it is preferred that women take only the recommended daily dose of 0.4 mg folic acid. In the countries, where 5 mg of folic acid is used, it is recommended to suspend folic acid supplementation for two weeks after taking IPTp with sulfadoxine–pyrimethamine to ensure optimal efficacy. Between 2010 and 2015, there was a five-fold increase in the percentage of women receiving the recommended three or more doses of IPTp in 20 African countries. To date, 36 African countries have adopted this policy and reported routine health facility data from the public sector on the number of women receiving the first, second, third and fourth doses of IPTp (i.e., IPTp1, IPTp2, IPTp3 and IPTp4). As of 2018, coverage rates of IPTp1, IPTp2 and IPTp3 were 60%, 49% and 31%, respectively [1]. Only Burkina Faso and the United Republic of Tanzania were estimated as having more than half of pregnant women receiving IPTp3 in 2018. The gap between high antenatal care (ANC) attendance and the low proportion of eligible pregnant women receiving IPTp3 largely reflects a failure of the health system to provide IPTp-SP at ANC facilities [31]. This is an economical–political aspect that should be considered and, hopefully, improved since it is demonstrated that IPTp decreases the incidence of low birth weight by 29%, severe maternal anaemia by 38%, and neonatal mortality by 31% [32].

Increasing parasite resistance to SP has led to evaluation of other combination therapies as potential alternatives [33,34]. Different clinical trials have shown the potential of ACTs as IPT, but at present no clear indication emerged to induce a change in policy. A meta-analysis considered 11 published randomised controlled trials or prospective cohort studies on the use of dihydroartemisinin (DHA)–piperaquine (DHA–PPQ). Among these, nine were IPT trials. The authors conclude that DHA–PPQ is safe, well tolerated, and more effective than other treatments, in terms of protection, for IPT [35]. However, the prolonged exposure needed for IPT needs further investigation on potential cardiac toxicity. Another very recent review and meta-analysis of DHA–PPQ versus SP for malaria prevention in pregnancy suggest that DHA–PPQ is more effective than SP in decreasing maternal and placental malaria [36].

## 4. Embryotoxicity of Artemisinins: An Overview of the In Vivo Studies

To better understand the results on the embryotoxicity of artemisinins in experimental animal models, a description of the morphogenesis of the circulatory system in these models is provided in the following sections.

### 4.1. Circulatory System Morphogenesis in Mammal Embryos

Heart morphogenesis: Heart progenitor cells are recognizable at the gastrula stage and aggregate to form two pair groups of cells constituting the cardiogenic areas. This cardiac field contains multipotent progenitor cells that will differentiate into hemangioblasts (the precursors of vessels and blood) or in multipotent cardiac precursors. The fusion of the pair heart primordia occurs in humans at 3 weeks of gestation and corresponds to the beginning of pulsation. Contemporaneously, blood vessels form independently [37,38].

Blood vessel formation: The development of blood vessels occurs by two temporally separated processes: vasculogenesis (the formation de novo of a network of blood vessels) and angiogenesis (consisting of the remodeling of the primary formed network to differentiate distinct capillaries, arteries and veins). During the first phase of vasculogenesis, hemangioblasts condense in aggregates called blood islands; in the second phase, the inner cells of blood islands are instructed to differentiate into blood cells, while the outer cells (angioblasts) will differentiate into endothelial elements that interact with smooth muscle-like cells (pericytes); in the third phase, finally, the endothelial cells of different tubes interconnect and form the primary capillary plexus. In amniotes (including mammals), vasculogenesis occurs firstly at the level of the yolk sac (extraembryonic vasculogenesis) and later at the level of the embryo itself (intraembryonic vasculogenesis) in order to form the aorta and the great vessels [39,40].

During angiogenesis, vascular endothelial growth factor (VEGF) instructs some endothelial cells (tip cells) to sprout to form new vessels toward the source of VEGF, distributed in the different organs, allowing the organ-specific capillary formation.

Hematopoiesis: As mentioned above, the primary sites of mammal’s hematopoiesis are the blood islands. Hematopoietic stem cells, originated at the level of yolk sac blood islands, seem to migrate and colonize the liver and the bone marrow to generate the adult hematopoietic stem cells. However, an intraembryonic production of hematopoietic stem cells (from the hemogenic endothelium at the level of the aorta–gonad–mesonephros, or AGM) has been demonstrated, as well.

### 4.2. Hematopoiesis in Amphibian (Xenopus) Embryos

In amphibians, the intermediate and definitive hematopoiesis is at the level of the hepatic marrow. In the *Xenopus* embryo, the liver is colonized by hematopoietic stem cells which originate, from the mesoderm of the ventral blood islands (homolog to the mammalian extraembryonic site) and from the dorsal lateral plate mesoderm (homolog to the mammalian intraembryonic sites).

### 4.3. Hematopoiesis in Fish (Zebrafish) Embryos

Zebrafish are an alternative model to evaluate angiogenesis since fish, like amniotes, develop yolk sac vasculature as the primary hematopoietic source. In Zebrafish, the primitive hematopoietic sites are the bilateral stripes of the lateral mesoderm, which converge medially to form the region where primitive erythrocytes are produced. After the onset of circulation (about 24 h postfertilization), definitive hemopoietic stem cells appear in the AGM region. These cells, 2–6 days postfertilization migrate and colonize the intermediate site of blood development (the caudal hematopoietic tissue, the thymus, and the kidney). In adulthood, the kidney marrow is the definitive primary site of hematopoiesis.

## 5. Developmental Toxicity Data of Artemisinin Derivatives

### 5.1. In Vivo Studies

**Figure 2:**
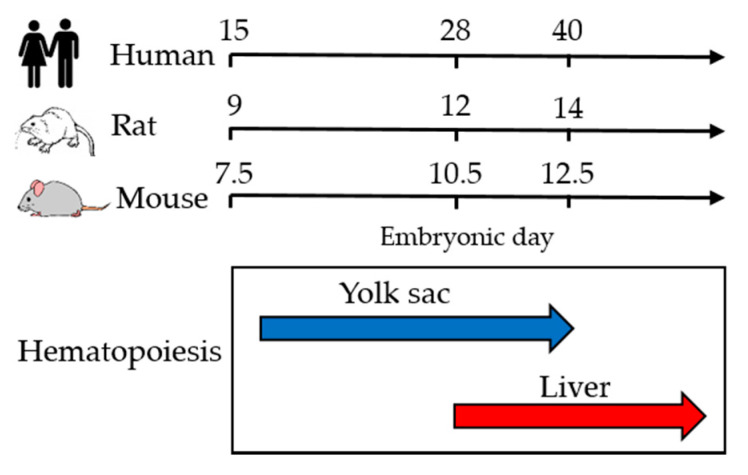
Phases of hematopoiesis in experimental rodents and humans during embryogenesis.

In this section, we will comment on the in vivo results from the most relevant studies describing the embryotoxic effects and malformations induced by artemisinin derivatives in different animal species. To better understand the results of this section, Figure 2 indicates the different phases of hematopoiesis in experimental rodents and humans during embryogenesis. Experimental toxicological data were obtained both after in utero exposure, by treating pregnant rodent females, and after in vitro exposure of embryos, using rodent postimplantation whole embryo culture (WEC) [41]. This model, widely used to study developmental toxicity, consists of culturing for about 48 h the entire embryo in vitro, excluding maternal factors from the system. One advantage is that mechanistic studies, such as drug–drug interactions, are more feasible. Dihydroartemisinin (DHA) was used in many in vivo studies since it is an antimalarial drug on its own and it is the main metabolite of other artemisinin derivatives used in human therapy. For instance, artesunate, the most broadly used artemisinin derivative, is readily hydrolysed to DHA both in animals and humans, as demonstrated also in pregnant women [42].

Developmental in vivo toxicity studies demonstrated that artemisinins can induce foetal death and congenital malformations in rodents only after embryo exposure within a narrow window in the early embryogenesis. Embryo deaths and malformations have been, in fact, observed in rats following oral administration of artesunate on single days between gestation day (GD) 10 and 14, while the same dose of the drug administered on GD 9 or a higher dose on GD 16 or 17 did not induce embryotoxicity. GDs 10–14 were consequently identified as the developmental period more sensitive to the artemisinins, with GD10 the most sensitive day to induced malformations (mostly cardiovascular defects and bone anomalies) and GD11 the most sensitive to induced embryolethality [43]. In other studies, the same pattern of teratogenicity was seen when different artemisinin derivatives (DHA, artesunate, arteether and artemether) were given at GD10, indicating that embryolethality and malformations were probably associated with the endoperoxidic bridge which characterizes the drug class and is responsible for the antimalarial activity [44]. This would exclude the possibility of developing artemisinin derivatives which maintain the antimalarial activity while reducing embryotoxic effects. However, endoperoxide artefenomel (OZ439) showed an improved safety margin compared to artesunate [45]. The authors hypothesized that artemisinins’ embryotoxicity is due to the reduction of heme biosynthesis and that artefenomel, due to its structure, is less active at this level. Another approach to decrease embryotoxicity of artemisinins is the association with other treatments which protect from toxicity. For example, folic acid administration in mice prevents the damages induced by artesunate in heart development, measured as septal defects, and the thickness of ventricular and atrial septa [46]. Drug conversion between the animal studies and human treatment indicate that the drug concentrations tested in these animal studies were within the range of the exposure doses during human treatment [47].

To better evaluate the pathogenetic mechanisms related to the artemisinins’ embryotoxic effects, Longo and colleagues [48], using the WEC method, showed that the yolk sac is the primary site highly susceptible to artemisinin compounds: a severe depletion of circulating erythroblasts was observed in rat embryos exposed in vitro during the whole culture period (GD9.5–11.5) to DHA. Similar results were observed when the embryos were exposed for 1.5 h at the beginning of the culture (GD9.5), while only effects on red blood cell morphology were seen after exposure for 1.5 h at the end of the culture period (GD11.5). The tested concentrations (0.01–2 µg/mL) were those in the range of maximal concentration detected in the plasma of patients after administration of the drug. It is relevant to consider that the WEC method covers a significant period of yolk sac hematopoiesis (primary hematopoiesis), which occurs from GD 9 to GD14 in rats and corresponds to the gestational period from GD 15 to week 6 in humans. After a single in utero exposure to DHA (treatment of a pregnant rat with 7.5 or 15 mg/kg), the same research group showed that, at GD 9.5 and 10.5, primitive erythrocytes from yolk sac hematopoiesis were affected, causing anemia and consequent hypoxia. Cell damage was evident in the embryo at GD 11.5 and, later, embryo–fetal death occurred [49]. In another study, the WEC model was used to analyse the mechanisms inducing depletion of the embryonic erythroblast. Abnormal cell division and apoptosis of the embryonic erythroblast after DHA treatment of embryos were demonstrated by the presence of increased symmetric and asymmetric binuclear cells, Terminal deoxynucleotidyl transferase dUTP nick end labeling (TUNEL)- and Caspase-3-positive cells and embryonic erythroblasts with fragmented nuclei [50].

Although the majority of the studies on artemisinins’ developmental effects are in rodents, alternative animal models such as the FETAX (Frog Embryo Teratogenesis Assay-*Xenopus*) were also used. A reduction of the primitive red blood cells were seen after the exposure of *Xenopus laevis* embryos to DHA (0.01–0.5 µg/mL) during the early developmental period (from 24 h post fertilization), whereas the red blood cells of older larvae were only minimally affected [51]. In this study, embryolethality did not occur and congenital anomalies were induced only at the highest dose level (0.1–0.5 µg/mL).

The zebrafish model was also used to test the potential embryotoxicity of DHA [52]. DHA exposure (1–10 µg/mL) during primitive hematopoiesis and hematopoietic stem cell specification caused an abnormal embryonic phenotype (pericardial edema, abnormal trunk axis, abnormal pigmentation), developmental delays or death, similarly to the other animal models. However, differently from the other animal models, DHA in zebrafish increased vasculogenesis and angiogenesis. This may be due to differences in culture conditions and drug delivery route in zebrafish compared to mammals, but it may also suggest a different mode of action. The fact that the hematopoietic sites are different in zebrafish vs. mammals must be taken in account.

Only one study was performed in primates. Artesunate showed a dose- and time-dependent developmental toxicity after treatment of 15 pregnant cynomolgus monkeys on GD 20–50 for different days intervals. The authors concluded that artesunate caused embryo-lethality at doses ≥12 mg/kg/day administered for more than 12 days at the beginning of organogenesis, but not when administered for shorter treatment period [53]. It is interesting to note that although ACT and not artemisinin monotherapy, is the recommended treatment against uncomplicated malaria, the developmental toxicity of ACTs, compared to artemisinin derivatives alone, has been scarcely studied in vitro [54,55].

### 5.2. In Vitro Studies

**Figure 3:**
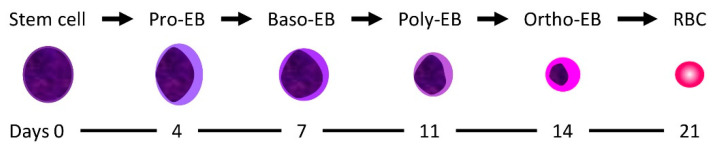
Erythroid development from the hematopoietic stem cell to mature erythrocytes (EB: erythroblast; baso: basophilic; poly: polychromatic; ortho: orthochromatic.

In vitro studies were performed from our group to characterize the target erythroid population in humans. As a model, human CD34+ stem cells from peripheral blood were chosen. DHA caused inhibition of cell proliferation and a delay of erythroid differentiation when it was added to the pro- and basophilic erythroblasts but not on mature erythroid stages (Figure 3). It seems indeed that DHA specifically target the pro-erythroblast and basophilic erythroblast stage when human CD34+ stem cells differentiate toward the erythroid lineage [56]. These data are in line with the animal studies described above, which suggest that embryotoxicity depends on the GD when artemisinins are administered, both for in utero and for the WEC studies. The experimental data from our group suggest that a window of susceptibility to artemisinins could occur also in human erythropoiesis.

As mentioned above, all the artemisinin compounds possess a distinctive and essential 1,2,4-trioxane pharmacophore. When artemisinin toxicity was studied in vitro against the K562 leukaemia cell line (a model for differentiating early human erythroblasts), it was found that the peroxide bridge was responsible for the inhibition of erythroid differentiation. All artemisinin derivatives used in the study were able to inhibit erythroid differentiation and, among different artemisinins, DHA was the most toxic. By contrast, the non-peroxidic deoxyartemisinin failed to inhibit the process [57]. Moreover, a different substituent in position C10 of the molecule can modulate the drug toxicity. Another in vitro study in fact indicated that artemisone, a novel 10-alkylamino derivative highly potent on *P.falciparum* [58], was less antiangiogenic than dihydroartemisinin, suggesting that it could be safer during pregnancy [59].

Taken together, in vitro human and in vivo animal models encourage the search for new artemisinin derivatives which maintain a fast antimalarial activity but show lower developmental effects in pre-clinical models.

## 6. Human Data on Artemisinin Derivatives: Embryotoxicity or Safety?

Pregnant women, especially in the first trimester of pregnancy, are frequently excluded from pharmaceutical trials for drug development because of the possible harm to the woman and/or the embryo/fetus. There are indeed limited available data regarding safety and efficacy profiles of most licensed antimalarials, including artemisinin derivatives, in pregnancy. Consequently, limited information is also available on the changes in ACT pharmacokinetics during pregnancy, especially in the first trimester.

Clinical studies evaluating the safety of ACTs treatments in the second and third trimester of pregnancy demonstrated that ACTs are safe for women and children [20]. Regarding the use of artemisinins in the first trimester of pregnancy, only observational study and not randomized controlled trials have been done to date.

Table 2 shows observational studies published from 1998 to 2020 reporting data on the association between the use of artemisinin derivatives in the first trimester and pregnancy outcome, including miscarriage, neonatal mortality, low birth weight (LBW), congenital malformations. The total number of women treated with artemisinin derivatives in these studies is 1045 (753 in Africa and 292 in Asia). Artemether/lumefantrine (AL) is the most used ACT in the studies on artemisinin safety in pregnancy. Few studies were conducted with monotherapy or other ACTs. In some studies, the specific type of ACT is not indicated.

As shown in Table 2, data on the safety of AL in the first trimester of pregnancy are reported in four studies conducted in Africa. The most recent study is by Augusto et al. in 2020 [60]. This paper analyzed the data coming from a multi-center prospective observational cohort study involving women at health and demographic surveillance sites in three countries in Africa: Burkina Faso, Kenya and Mozambique [74]. The study included 26 and 92 women exposed in first trimester to AL or quinine, respectively, and aimed to determine the association between drug exposure and low birth weight (LBW) and small for gestational age (SGA). The results indicate that AL exposure in the first trimester is not associated with an increased occurrence of LBW or SGA compared to non-exposed women. However, a higher prevalence of LBW and SGA was observed for children born to quinine-exposed pregnancies. The LBW prevalence among newborns was 10.9% and 26.9% among women exposed to ACT or quinine, respectively, compared to 9.5% among women unexposed to antimalarials. Quinine, but not AL exposure, was associated with adverse pregnancy outcome in another study in Tanzania that considered 319 women who assumed antimalarials in the first trimester [66]. The majority of these women, 172 (53%) used AL, the others used quinine (24.4%), sulfadoxine–pyrimethamine (20.7%) or amodiaquine (3.4%). Exposure to AL in the first trimester seemed indeed more common than other antimalarials. Increased risk of miscarriage and stillbirth was associated with the use of quinine and not with the other treatment including AL that resulted safer than quinine. The use of AL as a common practice in women in the first trimester of pregnancy was also reported in Uganda. Even if this study did not report the safety of the treatment, 98.6% of all 500 pregnancies (in all trimester) ended in a live birth and stillbirth occurred in seven cases [75].

Another observational study in Zambia [63] focused on first trimester exposures to AL considering data from a previous analysis of the full cohort of pregnant women in all trimesters of pregnancy, where it was observed that exposure to AL was not associated with perinatal mortality, malformations, or developmental impairment [76]. When focused on first trimester, 156 and 138 women exposed to AL or SP, respectively, were considered. Women in both groups had rates of preterm deliveries or LBW similar to those of women who did not receive treatments. Moreover, infant neurodevelopment up to 12 months was similar between the arms.

No significant increase in congenital defects was also observed in a prospective observational study in Rwanda. However, in this study, an increased frequency of obstetric adverse outcomes (abortion, peri-natal mortality, stillbirth and premature delivery) was observed after AL treatment during all trimesters of pregnancy [68]. In another observational study, in the first trimester of pregnancy, the risk of abortion was over 60% in women receiving an ACT (7/11) compared to 1% (1/38) in women treated with quinine. None of the 10 women who received intravenous artesunate miscarried. The authors assumed that the higher rates of abortion in women exposed to dihydroartemisinin–piperaquine (DHA–PQ) may reflect intrinsic biases within the non-comparative observational study. In this study prescription of DHA–PQ in the first trimester was reserved for those women who were more unwell, and this may explain the poor outcome [65].

Taken together, the ten studies conducted in Africa show that AL is the most common ACT treatment in the first trimester of pregnancy. The treatment is not teratogenic, nor does it increase the number of babies with low birth weight or small for gestational age, nor the stillbirths or the impairment in infant neurodevelopment and it seems safer than quinine.

The use of ACT other than AL is reported in six other studies in Africa (Table 2). In an observational study in central eastern Sudan, 62 pregnant women who had received artemisinin derivatives (48 received artemether injections, 11 artesunate plus sulfadoxine–pyrimethamine, 3 artemether plus lumefantrine) during the first trimester were followed-up until delivery and babies were followed-up until the first birthday. Two of the women treated with artemether injections miscarried while receiving quinine for a second malaria attack. The other 60 women who had received artemisinins delivered healthy infants at full term without congenital malformations and all infants survived in their first year of life. Moreover, no maternal deaths were recorded during the follow-up [69].

Although with limitations for the very low number of women involved, similar conclusions came from a recent prospective observational study in which thirteen women were identified who received artesunate–amodiaquine during the first trimester of pregnancy. Twelve women had experienced deliveries of live newborns with no congenital malformations. One woman had experienced a spontaneous abortion with a birth defect which was judged not to be related to ACT treatment, but rather to placental malaria [61].

Artesunate treatment in the first trimester of pregnancy is not as common and most of the studies have been done in Thailand [77,78]. The rates for abortion, stillbirth, congenital abnormality, and mean gestation at delivery did not differ from the rate of their communities.

Saito and colleagues performed a systematic literature review and meta-analysis considering 48 eligible efficacy studies, both observational and interventional cohort studies, including 7279 treated *P.falciparum* episodes of uncomplicated malaria in all trimesters of pregnancy [79]. Artemisinin based treatment showed higher efficacy than quinine treatment. However, the authors emphasize a high inhomogeneity in the design of studies, suggesting a standard framework for efficacy studies in order to obtain more comparable and reliable results [80]. More recently, 1242 papers with 5510 participants were included in a meta-analysis of safety of anti-malarial treatment in pregnant women. The results indicated significantly lower risks of abortion with quinine or AL compared to dihydroartemisinin–piperaquine, artesunate–mefloquine and artesunate–amodiaquine. However, no significant differences in the risk of stillbirths or neonatal deaths were observed with any of the drugs [81].

Studies on the use of antimalarial drugs during pregnancy and lactation, including the artemisinin derivatives, have been also reviewed by Gomes in 2016 [54] and by Saito and colleagues [11] in 2018. From these reports and the data shown in Table 2, it appears that ACT is more efficacious than monotherapy and well-tolerated in pregnancy and should be considered first-line for treating all pregnant and lactating women with uncomplicated *falciparum* malaria. However, the authors recommend that systematic monitoring for adverse effects is continued.

The Centers for Disease Control and Prevention (CDC) is more prudent in proposing the ACTs artemether–lumefantrine during the first trimester of pregnancy only when other treatment options (mefloquine or quinine plus clindamycin) are unavailable. Ballard and colleagues updated CDC recommendations after a literature review of 21 articles, which included one meta-analysis and five randomized open label controlled trials about the efficacy of ACT in uncomplicated malaria cases in pregnant women [82]. The authors analysed the results of sixteen studies on ACT safety in the second and third trimester, whereas for the first trimester they only considered the meta-analysis by Dellicour and colleagues that is described above [15].

All the studies reported above seem to indicate that artemisinins in different combinations or artesunate as monotherapy are safe even when administered in the first trimester of pregnancy. However, in the absence of randomized clinical trials, it is still difficult to draw definitive conclusions.

## 7. Differences between Animal Studies and Human Data

The differences observed between animal studies and human data about the safety of artemisinin derivatives in pregnancy are difficult to reconcile and can be attributed to different causes.

First of all, the animal studies are conducted in healthy animals, whereas the drug exposed women are infected with malaria parasites. Situation, pregnancy and malaria are known to influence the pharmacokinetic properties of several drugs, including antimalarials [83]. After oral administration of AS, the plasma concentrations of the parent drug AS and its main metabolite, DHA were higher in pregnant women with malaria compared with those in the same women in a healthy state namely in the postpartum period [42]. AS absorption and its conversion to DHA is different in malaria patients and in healthy volunteers [84]. Moreover, the stability of endoperoxide drugs can be different in different physiological situations. We demonstrated that the degradation of DHA in vitro is influenced by different assay conditions (pH, temperature, haemolysis) indicating that clinical disorders such as fever, haemolysis or acidosis associated with malaria severity may contribute to artemisinin instability [85]. Pregnancy may add further variability.

In malaria patients, artemisinins concentrate mainly on infected erythrocytes [86]. It has been proposed that, during malaria infection, artemisinins are less available to pass to the embryo since higher quantities are activated, and thus degraded, to form carbon-centered radicals which bind parasite proteins [54].

In rat and monkey embryos, the target of artemisinin toxicity was the circulating primitive erythroblasts [43,49,53]. Thus, considering that the primitive erythroblasts are predominant in the circulation of the human embryo between weeks 6 and12, it has been estimated that this is the most sensitive period in humans [53]. However, the treatment in humans with ACT lasts for a very short period (3–7 days) in the time window of greater susceptibility to artemisinin, when the proliferation of erythroblasts is very intense. Therefore, even if erythroblasts are damaged by DHA, they could be easily replaced by others of new formation and the toxic effects become marginal and with no clinical consequences [54,87]. The length of the exposure period of treatment appears thus, to be critical. Both data on safety in humans and data on embryotoxicity in animal models may be biased due to the time of exposure.

## 8. Conclusions

Are artemisinins safe in the first trimester of pregnancy? The lack of clinical trials specifically designed to answer to this question is a problem that cannot be easily solved due to ethical concerns. However, in the last few years, the subject was examined in more detail in clinical settings. As summarized in this review, several observational/comparative, prospective studies, and meta-analysis were conducted which have assessed the safety of using ACTs in the first trimester of pregnancy. The endorsement of the use of ACTs in the first trimester by the Malaria Policy Advisory Committee of WHO in 2015 was a first and important step towards the revision of WHO guidelines for the treatment of malaria in pregnancy that is still waiting to be implemented.

## Figures and Tables

**Table 1:** Recommended treatment for *P.falciparum* malaria during pregnancy (WHO, Guidelines for treatment of malaria).

**Treatment of Uncomplicated Malaria during Pregnancy**
*FIRST TRIMESTER* Quinine + Clindamycin for 7 days Quinine monotherapy if clindamycin is not available ACT is the alternative in case quinine + clindamycin is not available
*SECOND AND THIRD TRIMESTER* ACT: • Artemether + Lumefantrine • Amodiaquine + Artesunate • Dihydroartemisinin + Piperaquine
**Treatment of Severe Malaria during Pregnancy**
*ALL TRIMESTERS* Intravenous or intramuscular artesunate

**Table 2:** Observational studies on the use of artemisinin derivatives in the first trimester of pregnancy.

Author, Publication Year [ref]	ACT	Country	Period	N. of Women Involved	Period of Exposure	Conclusions
Augusto 2020 [60]	AL	Mozambique, Burkina Faso and Kenya	2011–13	92	2–13 weeks	No evidence of an increased risk of LBW, SGA or prematurity among pregnancies with ACT exposure during the first trimester of pregnancy
Rouamba 2020 [61]	ASAQ	Burkina Faso	2010–12	13	10 in the third month; 2 in the second; 1 in the first	12 women delivered live newborns (including one with twins) with no congenital malformations. One woman had experienced a spontaneous abortion that was judged not to be related to ASAQ
Moore 2016 [62]	ACTs	Thai–Myanmar border	1994–2013	183	5–11 weeks	No evidence that first-line treatment with an artemisinin derivative was associated with an increased risk of miscarriage or congenital malformations
Manyando 2015 [63]	AL	Zambia	2004–2008	156	<14 weeks	No evidence of higher risk of perinatal or neonatal mortality, premature delivery or low birth weight in women exposed AL compared with SP exposure
Dellicour S 2015 [64]	ACTs	Kenya	2011–13	77	6–13 weeks	Artemisinin exposure during the potential embryo-sensitive period was not associated with increased risk of miscarriage.
Poespoprodjo 2014 [65]	AS-DHA/PQ	Indonesia	2004–09	22		The risk of abortion was over 60% in women receiving an ACT compared to 1% in women treated with quinine. None of the 10 women who received IV artesunate miscarried
Mosha D 2014 [66]	AL	Tanzania	2012–13	172	<20 weeks	Exposure to AL in the first trimester was common. Quinine, but not AL exposure was associated with adverse pregnancy outcome
Dellicour 2013 [67]	ACTs	Senegal	2004–08	7	3 of 7 4–10 weeks	Exposure to ACTs resulted in normal live births.
McGready R 2012 [42]	Artesunate or ACT	Thailand	1986–2010	44	6–12 weeks	Risk of miscarriage was similar for women treated with CQ, Q or Artesunate
Rulisa S 2012 [68]	AL	Rwanda	2007–2009	96		Increased frequency of obstetric adverse outcomes but no significant increase in congenital defects after AL treatment in all trimesters of pregnancy
Adam 2009 [69]	Artemether-AS/SP-AL	Sudan	2006–2008	62	<12 weeks	Women delivered apparently healthy babies at full term. No congenital malformations, no preterm labour, no maternal deaths; none of the babies died during their first year of life.
Adam 2004 [70]	Artemether	Sudan	1997–2001	1	10 weeks	No abortion, stillbirth or congenital abnormalities in the newborn baby.
McGready 2001 [71]	Artesunate	Thailand	1992–2000	44	3–12.9 weeks	The rates of abortion, congenital abnormality, and stillbirth were all within the normal range of their communities
Deen 2001 [72]	Artesunate-PSD	Gambia	1999	77		No evidence of a teratogenic effect, no evidence of increased foetal loss or infant death
McGready 1998 [73]	Artesunate	Thailand	1992–96	13	3–12 weeks	No congenital abnormality in any of the newborn children, no adverse effect was found in women or neonates.
